# Dental Developmental Defects: A Pilot Study to Examine the Prevalence and Etiology in a Population of Children between 2 and 15 Years of Age

**DOI:** 10.3390/dj12040084

**Published:** 2024-03-25

**Authors:** Jorge Alvarado-Gaytán, Gloria Saavedra-Marbán, Laura Velayos-Galán, Nuria E. Gallardo-López, Manuel J. de Nova-García, Antonia M. Caleya

**Affiliations:** Department of Dental Clinical Specialties, School of Dentistry, Complutense University of Madrid, 28040 Madrid, Spain; alvaradojorge89@hotmail.com (J.A.-G.); gsaavedr@ucm.es (G.S.-M.); lvelayos@ucm.es (L.V.-G.); negallar@ucm.es (N.E.G.-L.); denova@ucm.es (M.J.d.N.-G.)

**Keywords:** dental development defects (DDDs), enamel development defects (DDE), modified enamel development defects index (DDEm), molar incisor hypomineralization (MIH)

## Abstract

Dental development defects (DDDs) are quantitative and/or qualitative alterations produced during odontogenesis that affect both primary and permanent dentition. The etiology remains unknown, being associated with prenatal, perinatal, and postnatal factors. The aims were to identify the possible etiological factors, as well as the prevalence of DDDs in the primary and permanent dentition in a pediatric population. Two hundred twenty-one children between 2 and 15 years of age, patients of the master’s degree in Pediatric Dentistry of the Complutense University of Madrid, were reviewed. DDDs were observed in 60 children. Next, a cross-sectional, case-control study was carried out (60 children in the control group and 60 children in the case group). The parents or guardians completed a questionnaire aimed at identifying associated etiological factors. The prevalence of DDDs in patients attending our master’s program in both dentitions was 27.15%. Otitis, tonsillitis, high fevers, and medication intake stood out as the most relevant postnatal factors among cases and controls. The permanent maxillary right permanent central incisor and the primary mandibular right second molar were the most affected; there were no differences in relation to gender. One out of three children who presented DDDs in the primary dentition also presented DDDs in the permanent dentition. Prenatal and postnatal etiological factors showed a significant relationship with DDD alterations, considered risk factors for DDDs in both dentitions.

## 1. Introduction

Dental developmental defects (DDDs) are alterations that happen during the stages of mineralization and in the process of amelogenesis, suggesting that the problem occurs during pregnancy and the first years of life [1,2]. Any alteration during enamel formation generates permanent changes because the ameloblast has little reparative capacity [3].

The first DDD that was given importance was the “mottled enamel” observed by McKay in 1901. This defect was related to excessive fluoride intake. Subsequently, defects with a clinical appearance different from that of mottled enamel became evident, which led to their classification into fluoride and non-fluoride enamel opacities. At this time, different classifications for DDDs emerged, generating confusion about their diagnosis. In 1992, the modified DDE index (DDEm) [4] of the FDI World Dental Federation (FDI) became more accurate and overcame many deficiencies detected in previous indexes. These indexes then classified DDDs into diffuse opacities, demarcated opacities, and hypoplasias [5].

The first research on one of the most frequently reported DDD, enamel hypomineralization, was carried out in Sweden in 1987. However, it was not until 2001 that Weerheijm et al. named this idiopathic hypomineralization of the enamel and suggested the terminology molar incisor hypomineralization (MIH) [6,7]. They defined it as a disorder of systemic origin involving one or more of the first permanent molars and often associated with opacities in the permanent incisors [8,9]. The severity of the lesion and the number of teeth with a DDD are related to the time at which the insult occurs. Research has also focused in recent years on primary molars, leading to reports of comparable lesions in hypomineralized second primary molars (HSPMs); Weerheijm et al. in 2003 stated that hypomineralization could also be found in second primary molars. The development of the second primary molars usually begins at the same time as that of the first molars and permanent incisors, with the maturation phase being slower in the first permanent molars (FPMs). For this reason, if the risk factor acts during this overlapping period, hypomineralization will affect not only the first molars and/or permanent incisors but also the second primary molar, receiving the latter the name hypomineralization second primary molars (HSPMs) or also called deciduous molar hypomineralization (DMH). The etiology of HSPMs seems to be more related to prenatal and perinatal factors. Since the second primary molars erupt 4 years earlier than the FPMs, having HSPMs may predict that the subject suffering from it may also present MIH years later [10].

Currently, the etiology of DDDs is unknown. Some of these theories suggest that risk or predisposing factors may be involved [10,11,12,13], which we could divide into (1) prenatal factors (episodes of maternal fever, prolonged medication, viral infections, preeclampsia, and maternal psychological stress [14]), (2) perinatal factors (prematurity, low birth weight, cesarean sections, prolonged labor, hypoxia, and complications during delivery [15]), and (3) postnatal factors (episodes of high fevers, respiratory problems, otitis, chickenpox, alterations in calcium and phosphate metabolism, exposure to dioxins due to prolonged breastfeeding, exposure to polychlorinated bisphenols, gastrointestinal alterations, antibiotic treatment (mainly amoxicillin), use of paracetamol and ibuprofen, subacute vitamin D deficiency and urinary infections and environmental factors [16,17]). The clinical appearance of DDDs is related to the stage of tooth formation at which the alteration occurs, the intensity, and the duration of the insult. Clinically, they can be observed as opaque areas with colors ranging from white/cream to yellow/brown. In demarcated opacities, the darker color has been associated with a lower degree of mineralization and higher protein content in the enamel [18,19]. DDDs have been associated with increased caries lesions, dental post-eruptive enamel breakdown (PEB), dental hypersensitivity, alterations in the patient’s behavior in the dental office, and even psychological alterations. It is important to mention that due to the clinical appearance of some DDDs, they are often confused with dental caries lesions, and it is necessary to know how to differentiate them to favor an adequate diagnosis and management of the alterations [18,19,20,21,22].

DDDs are a challenge for pediatric dentists and dentists due to their high and increasing prevalence [11,12] and the importance of knowing how to diagnose and treat them. Regarding the prevalence of DDDs, it is very variable. Thus, Wong et al. indicate that it can reach up to 89.9% of the population, while other studies, such as that of Jälevik et al., indicate that the prevalence drops to 33%. We also find authors who obtain intermediate data, such as Seow et al. who report an incidence of 58%. The comparison between the different studies is difficult to make because it depends on the population studied and the methodology used [23,24,25,26]. The aim of this study is to identify the possible etiological factors involved in DDDs in both primary and permanent dentition, as well as to estimate the prevalence in the pediatric population attending the master’s degree in Pediatric Dentistry at the Complutense University of Madrid.

DDDs are currently a challenge for pedodontics and dentists due to their high and increasing prevalence and the importance of knowing how to diagnose and treat them.

## 2. Materials and Methods

The present research was divided into two parts. First, a cross-sectional study was carried out to estimate the prevalence. Second, we conducted an analytical study of cases and controls.

### 2.1. Selection of the Sample

The sample included children between 2 and 15 years of age, residents of the Community of Madrid who attended their routine six-monthly checkups at the master’s degree in Pediatric Dentistry from the Complutense University of Madrid during the 2021–2022 academic year. In our postgraduate course, we treat children from all over the country, especially from the community of Madrid. Most often, they are very young patients who cannot be treated by the general dentist or patients with orofacial pathologies. A favorable report was obtained from the Ethics Committee of the Hospital San Carlos de Madrid (C.I.22/283-E 12) for the performance of the present study.

Prior to participation in the study, the parents or guardians of the child patients, as well as the patients themselves, were informed of the purpose and the voluntary nature of the study, signing a specific informed consent form for this purpose. All patients who could not undergo a complete intraoral examination, patients with orthodontic bands or incomplete deciduous dentition, children for whom the medical data could not be collected, and as well as adopted or foster patients were excluded. To ensure data protection, patient data were anonymized.

### 2.2. Sample Size and Sampling Approach

The EPIDAT 4.2 software was used to obtain the sample size. EPIDAT is a free software developed by the Epidemiology Service of the General Directorate of Public Health of the Health Department (Xunta de Galicia) with the support of the Pan American Health Organization (PAHO-WHO) and the CES University of Colombia. The calculation was made with an estimated prevalence of 25%; as mentioned above [23,24,25,26], the prevalence figures are very disparate. It was calculated with a precision of 5% and a confidence level of 95%. The target population was 500 patients, which was the number we had active in our master’s program, resulting in a sample size of 183 children. For the second part of the study, to investigate the etiology, we initially established 30 patients in the case group and 30 in the control group.

Finally, 221 children were screened, of whom 60 had DDDs. To investigate the etiology, 60 cases were included in the case group, and 60 cases without DDDs were randomly selected by a computer randomization system to form the control group. All patients who met the selection criteria and attended our master’s between October 2021 and March 2022 were included. A 6-month period was established because patients are reviewed every 6 months.

### 2.3. Evaluation Criteria

**Figure A1:**
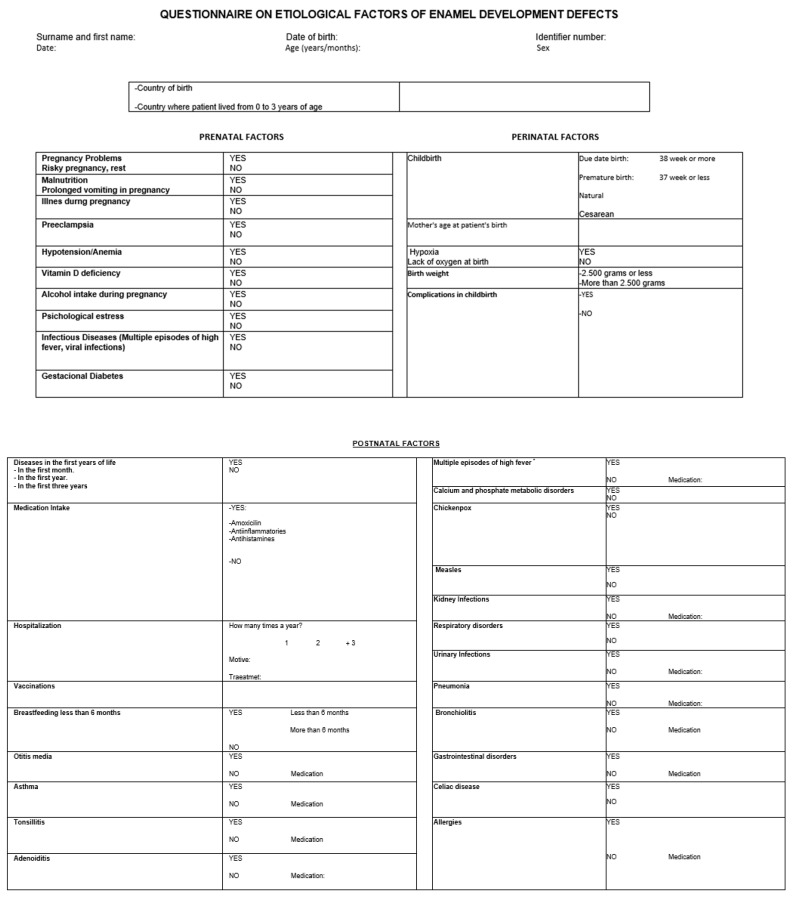
Questionnaire on etiological factors of DDDs.

The evaluation began with an initial examination of the participating patients in the facilities of the master’s degree in Pediatric Dentistry in a dental chair, using an artificial light source, and after drying all the teeth, the DDDs were recorded according to the categories proposed by FDI. Subsequently, a complete intraoral photographic series was taken (Nikon D100, 60 mm/10 mm f2. 8 sigma, Nikon macro speedlight sb-29s ring flash). In parallel, the parent or guardian of the patient, in the waiting room, filled out a written questionnaire of 27 items, mostly closed responses, in relation to the patient’s pre/peri and postnatal factors (Figure A1). The questionnaire was not validated, but it is based on an extensive literature review carried out for this research project [23,24,25]. This questionnaire was completed by the parents of the 60 patients with DDDs and by 60 randomly selected parents of patients without DDDs, The selection of the patients in the control group was randomly selected using OxMaR system (free software).

**Figure A2:**
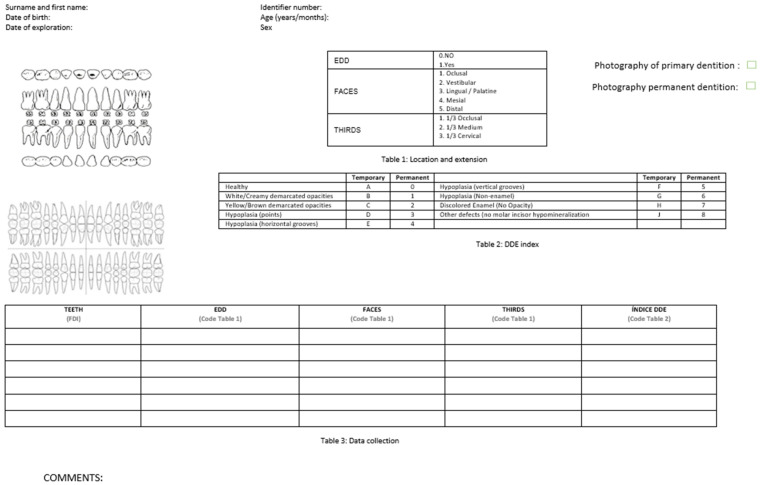
Data collection form.

A single, calibrated investigator oversaw the recruitment, examination, and taking of the patients’ photographic records. The investigator was calibrated with the FDI DDD photographic guide. The data corresponding to the DDD were collected on forms prepared for this purpose, guaranteeing the confidentiality of the patients through the application of the data protection law in force. Subsequently, the photographic images were viewed on a computer in full-screen mode, where the DDDs were reevaluated, and their classification was corroborated. To categorize DDDs, we based our categorization on the DDE index of the FDI World Dental Federation. The data collected can be found in Figure A2.

### 2.4. Statistical Analysis

Statistical analysis of the data was performed using the SPSS 28.0 program (IBM SPSS, 2021). Descriptive statistics of the quantitative variables were performed, and the Shapiro–Wilk normality test was applied. To contrast the influence between two qualitative variables, the Fisher’s exact test or chi-square test was used, and for the comparison of two means in quantitative variables, the Student’s *t*-test was used, assuming or not the equality of variances. Normality is assumed in the data. Equality of variances is tested with Levene’s test (which will indicate whether the test assuming equal or unequal variances is more appropriate). Regarding the comparison of quantitative variables between two groups, the nonparametric Mann–Whitney test was used. A statistically significant result was considered when *p* < 0.05.

## 3. Results

### 3.1. Prevalence of DDDs in Patients of the Master’s in Pediatric Dentistry of the Complutense University of Madrid

Once the selection criteria were applied, a population of 221 patients aged 2–15 years was obtained, with a mean age of 8.91 years. A total of 60 patients presented DDD alterations, forming part of the case group, and the rest of the population did not present any DDD alteration, from which 60 patients were chosen for the control group. The data from this study were obtained from a questionnaire completed by 120 participants. A total of 61 girls (50.8%) and 59 boys (49.2) between 2 and 15 years of age were included, with a mean age of 8.91 years. Out of all of these, 60 patients had DDDs.

The prevalence of DDD in patients attending our master’s program in both dentitions was 27.15%; the prevalence of DDDs in the permanent dentition was 20.36%, and in the primary dentition was 14.93%. In the case group, when Fisher’s exact test was applied to compare the percentages of the presence of DDDs in the primary dentition according to gender, no statistically significant differences were found at 95% (Fisher *p* = 0.123) in the primary DDDs between boys (66.7%) and girls (45.5%). As for the permanent dentition, Fisher’s exact test was also applied to compare the presence of DDDs in the permanent dentition and the patient’s gender. No statistically significant differences were found at 95% (Fisher *p* = 0.073) in the permanent DDDs between boys (63.0%) and girls (84.8%).

### 3.2. Distribution, Location, and Extent of DDDs

In the 60 patients detected with DDDs, a total of 373 teeth, 123 primary and 250 permanent, were checked. It was observed that 25% of the sample presented DDDs in the primary dentition, 45% in the permanent dentition, and 30% presented DDDs in both dentitions. Table 1 and Table 2 show the frequency of DDDs in permanent and primary teeth, respectively.

In the permanent dentition, 54.4% of the DDDs were recorded in the maxilla and 45.6% in the mandible. Likewise, 55.28% of the DDDs were found in the maxilla and 44.72% in the mandible in primary teeth. The most frequently affected surface in both dentitions was the buccal surface, followed by the occlusal surface. In addition, it was observed that the most frequent clinical signs of DDD in both dentitions were white/cream-colored opacity and hypoplasia in the form of dots (Table 3).

**Figure 1:**
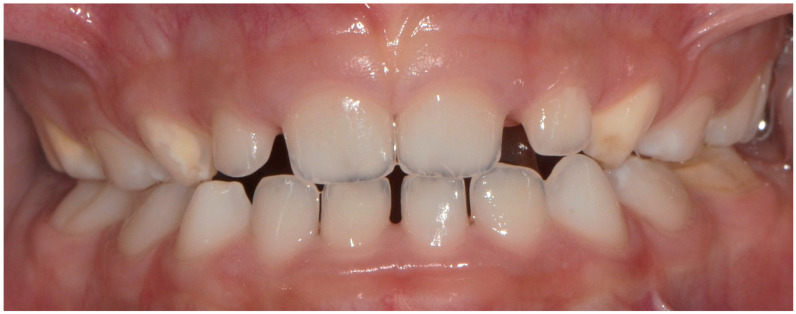
DDD in primary dentition localized in 55, 53, 63, 75.

**Figure 2:**
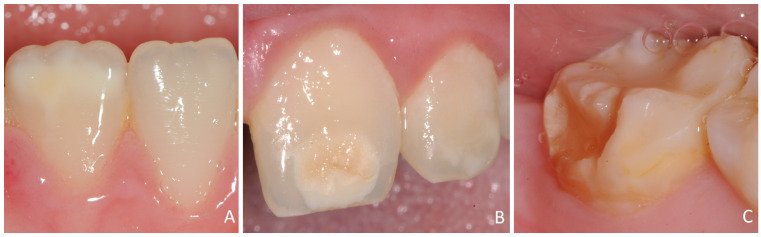
DDDs in permanent dentition: (**A**) white/creamy demarcated opacities, (**B**) yellow/brown demarcated opacities, and (**C**) hypoplasia (non-enamel).

Of all the children evaluated in this study who presented DDDs in the primary dentition, 30% also presented DDDs in the permanent dentition; that is, approximately one out of every three children who presented DDDs in primary teeth also presented DDDs in permanent teeth (Table 4). Figure 1 and Figure 2 show examples of DDDs in the primary and permanent dentition, respectively.

### 3.3. Aetiological Factors Present in the Sample

The results obtained, as well as the differences between the case group and the control group, can be seen in Table 5. It was not possible to assess the association between risk factors, calcium and phosphate disorders, measles, and celiac disease with the presence of DDDs since no patients suffering from these pathologies were recorded.

## 4. Discussion

To study the etiological factors of DDDs, the ideal is to carry out prospective longitudinal and cohort studies to obtain more precise data, thus reducing measurement and selection bias. Despite the advantages of this type of study, few studies were found in the literature reviewed due to their long duration, high economic cost, latency period, and the possible loss of the sample during the time the study is carried out. For all these reasons, this type of study was not chosen as the first option, even knowing that in cross-sectional studies, there may be forgetfulness or confusion in relation to the diseases suffered by the mothers or children, the taking of medication, when it occurred or for how long [19,27].

Several of the studies consulted were performed with a sample size like ours, such as Alaluusua et al., Tapias-Ledesma et al., and Velló et al. [28,29,30]. Assessing the prevalence of a specific pathology in the population is different from studying the etiological factors of that pathology. According to the literature reviewed, there are varying studies that exhibit variations in these aspects. Some studies employ a smaller sample size, such as this study, while others employ a larger sample size, such as Li et al. and Garca et al. [31,32]. Of the studies consulted the one with the largest sample size was that of Fagrell et al., with 17,000 participants [11]. It should be noted that studies with a larger sample size obtain more reliable results for correlations between the presence of DDD alterations, prevalence, and possible etiological factors.

The DDE index of the FDI World Dental Federation allows the study of hypoplasia, hypomineralization, and other enamel defects and is the one used in the present study. Other authors, such as Weerheijm in 2001, Ly in 1995, Alalausua in 1996, and Kemoli in 2008 have made their own indexes to carry out their studies, always using the DDEm index as a guide. Other studies have performed the diagnosis of the alterations following the criteria of the DDEm index [31,33,34,35,36,37]. Using the same index allows an adequate comparison between the different studies. There is controversy about whether to dry the tooth prior to the examination; in this study, at the time of the clinical examination, the teeth were dried beforehand using the triple syringe or cotton pellets, coinciding with other studies [14,33,35,37]. However, the European Academy of Paediatric Dentistry in 2003 made the recommendation not to dry them, and several studies have followed this suggestion [36,38,39,40].

In recent years, published studies have increasingly focused on the prevalence and etiology of DDDs. In this study, the overall prevalence of DDDs was 27.15%, the prevalence of DDDs in the permanent dentition was 20.36%, and in the primary dentition, it was 14.93%. Wong et al. state that the prevalence of DDDs can reach up to 89.9%. Other authors have studied the prevalence of DDDs and have concluded that it varies between 4% and 75% depending on the population studied and the criteria used for scoring. It is difficult to compare the prevalence between different studies because, depending on the population studied, there will be multiple differences in relation to environmental factors, genetic factors, and social factors, in addition to the different methods used in the research work. The prevalence found in this study is based on patients attending a specific pediatric dentistry clinic, where the prevalence results may be overestimated since patients with pathologies usually attend. Regarding the gender of the patient, we did not find statistically significant differences in the presence of DDDs in both the primary and permanent dentition, which is in agreement with other studies. Other studies observed a higher prevalence of DDDs in girls and others in boys [1,35,37,40,41,42,43].

Regarding the pattern of involvement and type of tooth affected, we found a similar frequency of involvement between incisors and molars, not coinciding with other studies where the involvement of molars was greater. We found that the tooth most affected in the permanent dentition was the upper right central incisor with 7.5%, followed by the lower right and left first molar with 7.2%, then the upper left first molar and upper left central incisor with 7.0% and the upper right first molar with 5.6%. Kusku et al. and Martinez et al. found that the right upper first molar is the most affected, and the least affected is the right lower first molar [14,38]. There are studies that found the same results as ours with respect to the permanent incisors [14,32,38,43,44]. There are various authors who state that the risk of presenting DDD alterations in incisors increases the more molars that are affected. Regarding primary dentition, in this study, it was found that the tooth most affected by DDDs disorders was the lower right second molar (5.4%), coinciding with the findings of the study by Lunardelli [45]. The relatively high prevalence of DDDs disorders in primary second molars is a common finding in different studies [10,46].

In this study, it was observed that, of the 250 permanent teeth evaluated, 54.4% of the DDDs were in the upper jaw and 45.6% in the lower jaw. Likewise, in the 123 primary teeth evaluated, 55.28% of the DDDs were in the upper jaw and 44.72% in the lower jaw. Several of the studies reviewed found the highest frequency of DDDs in maxillary teeth and other reviewed studies found that the most affected arch is the mandibular arch [36,42]. Other studies, in their results, found no difference in the frequency of DDD alterations between both arches [47,48]. Regarding the tooth surface most affected, in the primary and permanent dentition, we observed that it was the vestibular/buccal side, coinciding with other studies. The most affected third that we observed was the occlusal [32,47].

In terms of the alterations found, demarcated opacities were the most frequently presented; in terms of color, the most frequent was white/cream, coinciding with studies by Cruvinel et al., Correa Faria et al., and Martinez et al. [12,13,38]. Yellow/brown color opacity is the least frequent in this study, coinciding with the study by Martinez et al. Other authors, such as Lunardelli and Peres and Masumo et al., obtained results contrary to ours, observing that diffuse opacities were the most frequent [45,49].

We also observed that 30% of all the children who presented DDDs in the primary dentition also presented DDDs in the permanent dentition, approximately one out of every three children. This leads different authors to affirm that when DDD is present in both dentitions, it is more likely that the factors involved are prenatal and perinatal and not postnatal [6,10,46]. The DDD that has been extensively studied is the MIH. Weerheijm et al. stated that hypomineralization defects can also appear in the second primary molars and that the etiologic factors of these alterations may be the same, although at an earlier stage [46]. Studies affirm that children with a history of hypomineralized primary second molars should be considered patients at risk of presenting any DDDs disorder. However, very few studies have investigated this relationship [10,46]. The presence of DDDs increases the risk of caries and tooth wear because the defective enamel is thinner and retains more bacterial plaque. Therefore, early detection of these alterations can help to establish a prevention program and thus allow a better prognosis and long-term quality of life [46,50].

The etiology of DDDs is not entirely clear, and the possible etiological factors are controversial. Concerning the prenatal factors studied, we found that psychological stress was significantly related to DDD alterations and that the mothers in the control group had a higher frequency of preeclampsia compared to the cases. Similar results to our study were obtained by Ghanim et al. regarding psychological stress during pregnancy [3,46]. Regarding perinatal factors, we did not find any factors significantly related to DDDs. However, there are studies that claim that preterm infants are prone to severe diseases, which in turn can cause DDDs, and others have shown that low birth weight infants have a higher risk of presenting enamel opacities. Research has also been conducted on complications during delivery, cesarean section, and hypoxia. In a Dutch study on the possible etiological factors of DDDs, a high frequency of birth complications and infant respiratory disease was observed. Based on these findings, it was suggested that hypoxia might influence the mineralization of tooth enamel. However, Beentjes et al. found that there was no association between DDDs and these complications at delivery, as did the results in this study [51].

As for postnatal factors, we found that children who suffered illnesses during the first three years of life such as otitis, tonsillitis, adenoiditis, respiratory tract or gastrointestinal disorders, high fevers, taking medications such as anti-inflammatory drugs and anti-inflammatory drugs and amoxicillin together, had a significantly higher prevalence of DDDs compared to the control group. Alaluusua, in 2010, concluded that illnesses suffered during the first year of life were related to increased susceptibility to DDD alterations in children. Several studies show that the aforementioned factors are predisposing factors for the appearance of DDDs disorders [2,28,43,52]. The studies by Tourino et al., Allazzam et al., Mishra et al., and Souza et al. agreed with this research in stating that the intake of medications in early childhood increases the risk of presenting DDDs. The study conducted by Biondi et al. considers that what is related to DDDs is only the intake of anti-inflammatories and not the intake of antibiotics during early childhood. Allazam et al. and Souza et al. agree with this study by finding a statistically significant relationship between children who presented multiple episodes of high fever in the first three years of life and the risk of suffering DDD alterations. Jankovic et al. are of the opposite opinion and affirm that episodes of fever are not related to the appearance of DDDs [1,16,17,39,43,50,53].

One of the limitations of our study is the calculation of prevalence. When the prevalence is calculated among patients attending a specific dental service, there may be an overestimation. Other limitations would be the sample size and the questionnaire used, which, despite being comprehensive and based on the literature, is not validated. On the other hand, the size of the control group should be larger in order to determine homogeneity criteria. However, we consider that due to the large number of studies on this subject, it was not necessary since we have incorporated these articles in the discussion of the manuscript. In subsequent studies, we recommend homogenization of the groups. As we have already mentioned, the overestimation produced by children attending a specific dental service, together with the biases that can occur in the collection of data by means of a questionnaire, can mean that the data cannot be extrapolated to the general population, since the main objective was to determine factors associated with DDDs and to determine the prevalence in our service as a secondary objective. However, the data obtained in our pilot study can serve as a starting point for a large multicenter prevalence study.

Some challenges were considered that could have been sources of detriment to the results. Group of patients from the master’s degree in Pediatric Dentistry at the Complutense University of Madrid, an institution to which they are included in control and follow-up plans due to their clinical pathologies. In our sample, the prevalence of DDDs was 27.15%. As researchers, we should never forget the clinical importance of our research. This means that in the case of HSPMs and MIH, cross-sectional studies are a good formula for recording epidemiological prevalence data. However, more longitudinal studies are needed that may be appropriate for establishing causal inferences, which are so important in the study of these conditions. Due to the high prevalence of DDDs in this study and another recent study carried out in children in the community of Madrid on the prevalence of other DDDs, such as molar incisor hypomineralization [54], we believe that further studies are needed and recommend looking at the site: https://www.thed3groups.org (accessed on 21 December 2023). The D3 Group (D3G) comprises a translational research and education network spanning the Developmental Dental Defect (DDD = D3) sector, originally in Australia and New Zealand and increasingly around the world [55].

Despite the different investigations, the etiology of DDDs is still unclear, and studies continue to be carried out since it is a multifactorial and complex disorder.

## 5. Conclusions

The prevalence of DDDs in the population studied was 27.15% in both dentitions; the prevalence of DDDs in the primary dentition was 14.93%, and in the permanent dentition was 20.35%.

As for the etiological factors involved in DDDs, in the present study, the most important prenatal factors were the psychological stress of the mother, and none of the perinatal factors studied were associated with DDDs. Of all the postnatal factors studied, children who suffered illnesses during the first three years of life and had a history of otitis, took medications such as anti-inflammatory and anti-inflammatory and amoxicillin together, a medical history of tonsillitis, adenoiditis, multiple episodes of high fever, chickenpox, respiratory tract infections, bronchiolitis or gastrointestinal disorders showed a significantly higher prevalence of DDDs disorders.

The presence of DDDs in the primary dentition is a predictor of the future presence of DDDs in the permanent dentition. Despite the different investigations, the etiology of DDDs is still unclear.

## Figures and Tables

**Table 1:** Frequency of DDDs in permanent teeth.

Teeth	Frequency	Percentage
**11**	28	7.5%
**12**	11	2.9%
**13**	2	0.5%
**14**	3	0.8%
**15**	1	0.3%
**16**	21	5.6%
**17**	1	0.3%
**21**	26	7.0%
**22**	9	2.4%
**23**	2	0.5%
**24**	4	1.1%
**25**	2	0.5%
**26**	26	7.0%
**31**	14	38.0%
**32**	9	2.4%
**33**	2	0.5%
**34**	3	0.8%
**35**	1	0.3%
**36**	27	7.2%
**37**	1	0.3%
**41**	8	2.1%
**42**	11	2.9%
**43**	4	1.1%
**44**	5	1.3%
**45**	1	0.3%
**46**	27	7.2%
**47**	1	0.3%
**Total**	250	67.0%

**Table 2:** Frequency of DDDs in primary teeth.

Teeth	Frequency	Percentage
**51**	3	0.8%
**52**	3	0.8%
**53**	8	2.1%
**54**	4	1.1%
**55**	16	4.3%
**61**	3	0.8%
**62**	3	0.8%
**63**	7	1.9%
**64**	7	1.9%
**65**	14	3.8%
**73**	6	1.6%
**74**	4	1.1%
**75**	14	3.8%
**83**	7	1.9%
**84**	4	1.1%
**85**	20	5.4%
**Total**	123	33.0%

**Table 3:** Face, thirds, and classification of DDDs, as well as frequency and percentage in the permanent and primary dentition.

Characteristics		Frequency (Permanent/Primary)	Percentage (Permanent/Primary)
**Surfaces**	Occlusal	116/81	46.4%/65.9%
	Vestibular/Buccal	230/113	92.0%/91.9%
	Lingual/Palatal	62/43	24.8%/35.0%
	Mesial	45/16	18.0%/13.0%
	Distal	41/16	16.4%/13%
**Thirds**	1/3	239/111	95.6%/90.2%
	2/3	103/66	41.2%/53.7%
	3/3	41/27	16.4%/22.0%
**Índex** **DDD**	White/Creamy demarcated	189/99	75.6%/80.5%
	Yellow/Brown demarcated opacities	70/36	28.0%/29.3%
	Hypoplasia (points)	113/60	45.2%/48.8%
	Hypoplasia (horizontal grooves)	113/51	45.2%41.5%
	Hypoplasia (vertical grooves)	26/6	10.4%/4.9%
	Hypoplasia (non-enamel)	26/16	10.4%/13%
	Discolored enamel (no opacity)	0/0	0%/0%
	Other defects (no MIH)	67/59	26.8%/48.8%

**Table 4:** Frequency distribution of DDDs.

	Frequency	Percentage	Valid Percentage	Cumulative Percentage
Primary DDDs	15	25	25	25
Permanent DDDs	27	45	45	70
Primary and Permanent DDDs	18	30	30	100
TOTAL	60	100	100	

**Table 5:** Description of etiological factors. The Fischer test and Pearson’s chi-square test were used for factor analysis.

	Etiological Factors	Frequency Group Cases	Frequency Group Cases	Frequency Control Group	Frequency Control Group	P T.Fisher Value	P T.Chi Square Value
**Prenatal**	Malnutrition	5	8.3%	11	18.3%	0.178	0.107
	Pregnancy problems	17	28.3%	17	28.3%	1.000	1.000
	Illness during pregnancy	8	13.3%	11	18.3%	0.618	0.453
	Preeclampsia *	**1**	**1.7%**	**10**	**16.7%**	**0.008**	**0.004**
	Hypotension/Anemia	8	1.3%	10	16.7%	0.799	0.609
	Vitamin D deficiency	9	1.0%	7	11.7%	0.789	0.591
	Alcohol intake during pregnancy	1	1.7%	0	0%	1.000	0.315
	Psychological stress *	**12**	**20.0%**	**2**	**3.3%**	**0.008**	**0.004**
	Infectious diseases	2	3.3%	0	0%	0.496	0.154
	Gestational diabetes	3	5.0%	4	6.7%	1.000	0.697
**Perinatal**	Due date birth	53	88,3%	53	88.3%	1.000	1.000
	Premature birth	7	11.7%	7	11.7%	1.000	1.000
	Natural birth	44	73.3%	42	70.0%	0.840	0.685
	Cesarean birth	16	26.7%	18	30.0%	0.840	0.685
	Hipoxia	8	13.3%	3	5.0%	0.204	0.114
	Birth complications	19	31.7%	12	20.0%	0.210	0.144
	Low birth weight (<2500 g)	9	15.0%	5	8.3%	0.394	0.255
	Normal birth weight (≥2500 g)	51	85.0%	55	91.7%	0.394	0.255
**Postnatal**	Diseases in the first month of life	10	16.7%	6	10.0%	0.421	0.283
	Diseases in the first year of life	15	25.0%	10	16.7%	0.369	0.261
	Diseases in the first three years of life *	**14**	**23.3%**	**5**	**8.3%**	**0.043**	**0.024**
	Medication intake * **(amoxicillin/anti-inflammatories/antihistamines)**	29	48.3%	5	8.3%	--	0.001
	Hospitalizations once a year	16	26.7%	16	26.7%	--	1.000
	Hospitalizations twice a year	3	5.0%	3	5.0%	--	1.000
	Vaccinations	60	100%	60	100%	1.000	0.315
	Breastfeeding less than 6 months	20	33.3%	15	25.0%	0.422	0.315
	Breastfeeding more than 6 months	36	60.0%	36	60.0%	1.000	1.000
	Otitis *	**26**	**43.3%**	**5**	**8.3%**	**0.001**	**0.001**
	Asthma	2	3.3%	5	8.3%	0.439	0.243
	Tonsillitis *	**15**	**25.0%**	**3**	**5.0%**	**0.004**	**0.002**
	Adenoiditis*	**10**	**16.7%**	**0**	**0%**	**0.001**	**0.001**
	Multiple episodes of high fever *	**16**	**26.7%**	**2**	**3.3%**	**0.001**	**0.001**
	Calcium and phosphate metabolic disorders	0	0%	0	0%	--	--
	Chickenpox *	7	11.7%	0	0%	0.013	0.006
	Measles	0	0%	0	0%	--	--
	Kidney infections	1	1.7%	0	0%	1.000	0.315
	Respiratory tract disorders *	**12**	**20.0%**	**4**	**6.7%**	**0.058**	**0.032**
	Urinary tract infections	4	6.7%	2	3.3%	0.679	0.402
	Pneumonia	4	6.7%	1	1.7%	0.364	0.171
	Bronchiolitis *	**20**	**33.3%**	**8**	**13.3%**	**0.017**	**0.010**
	Gastrointestinal disorders *	**15**	**25.0%**	**2**	**3.3%**	**0.001**	**0.001**
	Celiac disease	0	0%	0	0%	--	--
	Allergies	13	21.7%	8	13.3%	0.337	0.230

* Factors with statistically significant differences between the case group and the control group. In bold, we can see the differences between the possible etiologic factors between the cases and the control.

## Data Availability

Data to support the conclusions of this study are made available through the corresponding author, A.C.
